# Blood supply continuity and emergency preparedness in natural and man‐made disasters and in armed conflicts—The Nordic perspectives

**DOI:** 10.1111/vox.70242

**Published:** 2026-03-17

**Authors:** Agneta Wikman, Christian Medby, Denise Bäckström, Jarkko Ihalainen, Markus Lyyra, Patrik Nimberger‐Hansson, Geir Strandenes, Jouni Lauronen, Torunn Apelseth

**Affiliations:** ^1^ Department of Clinical Immunology and Transfusion Medicine Karolinska University Hospital Stockholm Sweden; ^2^ Department of Medicine Huddinge Karolinska Institutet Stockholm Sweden; ^3^ Swedish Armed Forces Stockholm Sweden; ^4^ Norwegian Armed Forces Joint Medical Services Sessvollmoen Norway; ^5^ Department of Trauma St. Olavs University Hospital Trondheim Norway; ^6^ Department of Biomedical and Clinical Sciences Linköping University Linköping Sweden; ^7^ Department of Leadership and Command & Control Swedish Defence University Karlstad Sweden; ^8^ Finnish Red Cross Blood Service Vantaa Finland; ^9^ Centre for Military Medicine Finnish Defense Forces Helsinki Finland; ^10^ Department of Anaesthesiology and Intensive Care Linköping University Hospital Linköping Sweden; ^11^ Norwegian Centre for Blood Preparedness Bergen Norway; ^12^ Department of Immunology and Transfusion Medicine Haukeland University Hospital Bergen Norway; ^13^ Department of Clinical Science, Faculty of Medicine University of Bergen Bergen Norway

**Keywords:** blood preparedness, blood supply, disasters, emergencies, war

## Abstract

Recent events have highlighted the urgent need for comprehensive blood preparedness plans at local, regional, national and cross‐border levels within the Nordic countries. This article outlines the perspectives and strategies related to blood preparedness in Norway, Sweden and Finland, in the context of emergencies, disasters and armed conflicts. Norway, Sweden and Finland share long borders and similar geography, including vast rural areas with limited health care resources. Finland operates a centralized blood service, whereas Norway and Sweden maintain decentralized systems, with regional authorities responsible for their own blood supply. All three countries adhere to a total defence concept, wherein military forces depend on civilian transfusion services. In mass casualty events, the demand for transfusion services to provide urgent blood and blood components is expected to be high. Haemorrhage remains the leading cause of preventable prehospital death in trauma cases, and the availability of blood products in prehospital settings is critical in both civilian and military environments. Key priorities identified include: establishing stockpiles of shelf‐stable blood products, developing surge capacity plans for rapid upscaling of blood collection and whole blood and component production, conducting joint training and exercises involving blood establishments, transfusion services, hospitals and military units, and collaboration in a pilot cross‐border initiative—the *Nordic Blood Preparedness Project*—in northern Norway, Sweden and Finland, aimed at harmonizing regulations and procedures during emergencies. To effectively save lives during disasters and armed conflicts, it is essential to have well‐documented and operational blood preparedness plans in place.

**Highlights**

- Availability of blood product as close as possible to bleeding patients saves lives.
- Local, regional, national and cross‐border blood preparedness plans are needed.
- Blood banks should participate in hospital, regional and relevant military exercises.

## BACKGROUND

The Nordic countries Norway, Sweden and Finland share long common borders and similar geography, with sparsely populated areas and few hospitals in the north and more densely populated areas in the south. Natural disasters such as earthquakes, floods and volcanos are rare, but there have been terrorist attacks and school shootings with large numbers of injured individuals. This has caused recognition of the need for preparedness and coordination in the health care sector and within the blood supply system [1]. The Russian full‐scale invasion in Ukraine has led to increased focus on both civilian and military preparedness, cross‐border collaboration and collective defence. All the Nordic nations rely on ‘total defence concepts’, where the military, civilian and private sectors all can be mobilized to support national or collective defence [2]. Since 2024, all Nordic nations are North Atlantic Treaty Organization (NATO) members, as well as our close neighbours around the Baltic Sea, Estonia, Latvia and Lithuania. The prospect of large‐scale combat operations in Europe is no longer considered an impossibility. Requests for blood support to conflict areas may arise within the NATO alliance (Figure 1) [3, 4].

**FIGURE 1:**
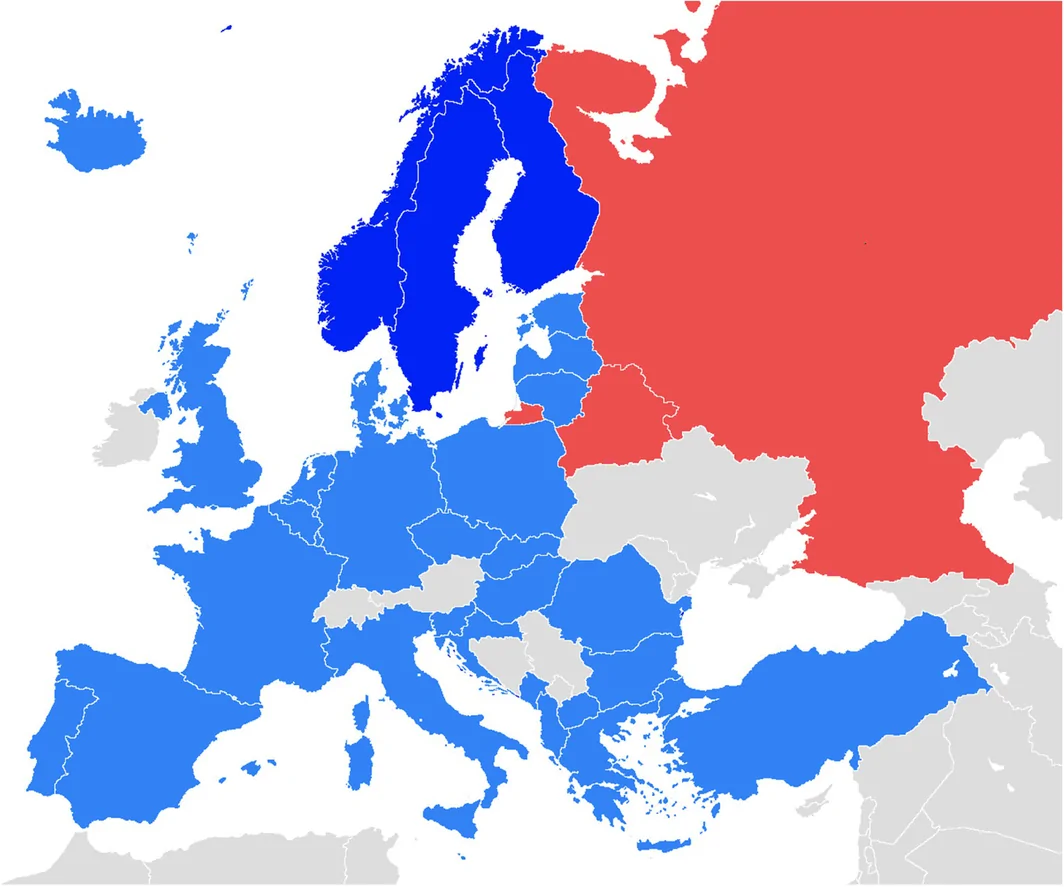
A map of North Atlantic Treaty Organization (NATO) members in Europe (light blue), with the Nordic countries (dark blue), non‐members (grey) and Russia and Belarus (red) (modified from San Jose/Wikimedia Commons).

Experience with mass casualty events in the Nordic countries has so far been limited primarily to incidents of terrorism. In Norway, the Utøya attack in 2011 by a far‐right extremist is the most severe violent mass casualty event in the country. The attack was initiated with a vehicle‐borne improvised explosive device in central Oslo followed by a mass shooting on the island, Utøya, where a youth camp for a political party was held. Seventy‐seven people were killed [5]. Experience from mass casualty events in Sweden comes from two attacks defined as terrorist attacks in the centre of the capital, Stockholm. One attack was carried out in 2017 using a truck driving into a pedestrian street, resulting in 5 dead and 14 injured; the other was a bomb attack in 2010 that resulted in 2 injured and the perpetrator's death [6, 7]. Sweden has also had two severe attacks in schools: in 2015, a former student attacked with a sword and killed 3 individuals before he was stopped [8] and in 2025, a former student killed 10 individuals with a gun before killing himself [9]. In 2002 in Finland, a bomb attack in a shopping mall killed 7 and injured 160 individuals [10]. Finland has also had several school attacks, with shootings or stabbings by former students [11, 12, 13].

A main challenge in these attacks was that access to the victims was delayed, to provide security for the rescue teams, which delayed initiation of treatment [14]. The lessons learned from these incidents as well as experience from other high‐profile attacks, such as in Boston, Paris, Brussels, Nice and London, is that many died because of prehospital bleeding, while mortality was low after arrival at hospital [5, 15]. Immediate blood supply is required to save lives when the patient is in haemorrhagic shock. Preparedness planning must take logistics related to transportation of blood, communication, information, blocked telephone lines, traffic jams and routines to provide prehospital blood into consideration [16, 17, 18].

In the Nordic countries and across Europe, blood legislation is currently harmonized under the European Union (EU) Directive 2002/98/EC, which sets standards for the quality and safety of human blood and blood components [19]. This directive will be replaced by the new Regulation on Substances of Human Origin (SoHO) from August 2027 [20]. The SoHO Regulation includes requirements for contingency planning and emergency preparedness in relation to blood supply. Additional guidance and detailed requirements are provided in the 22nd edition of the Guide to the Preparation, Use and Quality Assurance of Blood Components and Blood Supply Contingency and Emergency Plan, published by the European Directorate for the Quality of Medicines & HealthCare (EDQM) and guidelines published by World Health Organization (WHO) [21, 22, 23]. Previously, emergency preparedness efforts were primarily focused on pandemics, such as COVID‐19. However, due to on‐going wars, there has been a shift toward planning for large‐scale emergencies involving mass casualties and military conflicts.

The aim of this article is to describe the perspectives and strategies of blood preparedness for disaster and war in the Nordic countries, Norway, Sweden and Finland.

## STATUS TODAY

### Blood organization

Finland has a centralized blood organization preparing blood products for the whole country, while Sweden and Norway have decentralized organizations with the regions responsible for their own blood supply. In all three countries, most of the collected whole blood (WB) is used to produce blood components: red blood cells (RBCs), plasma and platelets. During mass casualty events, blood support from other hospitals may be needed. In Sweden and Norway, this has until recently been managed by the hospitals contacting each other. In Finland, hospitals order all blood products from the Finnish Red Cross Blood Service (FRCBS), which has stocks in different locations.

In Norway, a government‐funded national coordination centre for blood preparedness, the Norwegian Center for Blood Preparedness, was founded in 2022 [24]. The mission of the centre is to ensure an adequate and sustainable supply of blood products in times of crisis and war, establish a system that ensures civilian‐military coordination of the blood supply and strengthen preparedness for major civil disasters and in geographically isolated areas. The Norwegian Medical Products Agency is the competent authority for regulation regarding blood, cells and tissue.

In Sweden, the National Board of Health and Welfare Authority is responsible for the preparedness and coordination of blood supply between the regions. This includes leading, coordinating and supporting health care regions in disasters [25]. The aim of the manuscript was not to compare centralized versus decentralized organizations, but rather to describe the work of blood preparedness we are doing, working closely together. We have different blood organizations and preparedness plans and through our on‐going collaboration we are continuously mapping the differences and potential challenges to improve and facilitate cross‐border collaboration on blood preparedness. The aim is to harmonize routines to be able to support each other.

### Blood products and surge plans

Early balanced transfusion is recommended in the treatment of patients with major bleeding [26, 27]. In hospitals in the Nordic countries, this policy is adapted and achieved by using massive transfusion packages consisting of RBCs, plasma and platelets in a physiological ratio [28, 29]. In Norway, WB can be used as an alternative to blood components [30]. Hospital blood stocks and disposables are planned based on peace‐time demand, while aiming for low wastage of the blood components with limited shelf life. Rural hospitals often have a small blood stock of RBCs and fresh frozen plasma (FFP), but no thawed plasma and few or no platelets. In cases of unexpected bleeding, this can be critical even if only one patient is bleeding. Recently, low‐titre blood group O WB (LTOWB) in the treatment of bleeding has been introduced in some hospitals [31, 32, 33].

There is emerging evidence that time to blood resuscitation is important in bleeding patients and that blood products within less than 1 h may save lives [34, 35, 36]. Pre‐hospital blood is increasingly requested today and is provided to the ambulance helicopters in Norway and Sweden as LTOWB or RBCs and dried plasma (DP) [37]. In Finland, the ambulance helicopters carry RBCs and dried or thawed plasma, and two air ambulance helicopters and one ground ambulance carry WB as part of an on‐going study, FinnPHWB (ClinicalTrials.gov NCT05744583).

But still, the ground‐based ambulance service most often only have crystalloids as resuscitation fluids [38].

In Norway with many remote areas and often unfavourable weather at the Atlantic and Barents Sea coast, collection of WB for emergencies have been implemented in health care facilities in isolated places in the north and on off‐shore oilrigs, referred to as civilian Walking Blood Banks [39]. Briefly, LTOWB is collected from pre‐screened emergency blood donors for immediate transfusion to patients with severe bleeding awaiting evacuation. The programme includes regular testing and interviews of prescreened emergency blood donors, training of health personnel in emergency blood collection and transfusion, planning for material and equipment, revision and regulatory approval. The blood donors are tested every 6 months and post transfusion. Cold‐stored platelets where the shelf life is extended up to 14 days, are used in two hospitals [40, 41]. As stated by the Norwegian Surgeon General, the armed forces require LTOWB and dried plasma during combat operations, which are expected to be provided from the civilian transfusion service [42].

In Sweden, the lack of platelets in remote hospitals has been identified as critical and therefore priority has been on the development and evaluation of cryopreserved platelets [43, 44, 45, 46]. They have been extensively evaluated in vitro, can be stored for several years and are rapidly thawed and reconstituted in plasma at request [47]. Cryopreserved platelets have been used for more than 50 years, the last decade mainly on bleeding indications, and are assessed as safe and clinically effective [48, 49, 50]. They are now implemented and used in many remote hospitals, with previously no or a few platelet units in stock, and as backup inventory in larger hospitals in Sweden [51]. In a recently published randomized controlled trial (RCT) in Australia and New Zealand evaluating non‐inferiority versus fresh platelets in‐hospital in thoracic surgery, they have been shown to result in higher blood loss and more transfusions than fresh platelets but were not associated with safety issues and adverse events [52].

Freeze‐dried plasma is used in ambulance helicopters, some remote locations (oil rigs and rural municipal health care services in Norway), and by the armed forces. It has also been implemented in a few emergency hospital departments due to the long thawing time of FFP.

### Emergency plans, information technology (IT) systems and labels

Emergency plans are required for all blood banks and hospitals. However, these plans are often limited to a telephone alert chain and contacting larger hospitals (in Norway and Sweden) to request transport of group O or O RhD‐negative RBCs and platelets. In Finland, blood products are requested from the FRCBS. Some blood banks have surge plans that include calling in blood donors for urgent donations [53]. Very few hospitals currently have the capacity to provide WB in emergency situations.

Cyberattacks targeting IT systems are increasingly frequent in our countries. Ensuring secure laboratory information systems (LIS) is therefore a top priority. All transfusion services maintain backup systems, with manual procedures serving as the final fallback option in the event of system failure.

The International Society of Blood Transfusion (ISBT) 128 system for labelling blood products is used across the Nordic countries. However, there is significant variation in the barcodes and component/product names used. ISBT codes for blood components and products differ not only between countries but also between regions within the same country. Additionally, the clear text labelling appears in different languages. In emergency situations, it is essential that procedures for the import and export of blood products are well understood and clearly defined.

### Legislation and regulatory requirements

Despite shared legislation under the blood directive [19] national interpretations and requirements—such as testing and donor eligibility criteria—still vary. For example, Finland has required nucleic acid testing (NAT) for hepatitis B, hepatitis C and human immundefiency virus since 2008, whereas Sweden and Norway do not currently mandate NAT testing. As a result, the direct import of blood from Sweden and Norway to Finland is not permitted during peace time. Additional legal and logistical barriers—such as differing donor acceptance criteria, national personal identification systems and import/export regulations—are currently under discussion to facilitate future cross‐border blood supply agreements.

### Nordic collaboration

The Nordic countries maintain close military collaboration, both regionally and within NATO. The armed forces request WB or WB equivalents from civilian blood services. WB equivalents refer to RBCs, plasma and platelet units provided in WB proportions, with requirement of different transport and storage conditions. Within NATO, there is a shared ambition to establish interoperable blood support systems for military operations [3]. On the civilian side, Nordic health care cooperation occurs at multiple levels. Intergovernmental activities are coordinated through the Nordic Council of Ministers (www.norden.org), and the Nordic Health Preparedness Agreement—established in 2002—provides a framework for collaboration in health emergencies.

## STRATEGIES AND ACTIVITIES

In collaborative projects and discussions critical issues in blood preparedness scenarios have been identified (Table 1).

**TABLE 1:** Needs and activities of blood preparedness (Substances of Human Origin Regulation European Union [EU] 2024/1689, articles 63‐67).

Needs	Activities	Responsible
Timely available blood products in bleeding situations	Implement guidelines of required blood inventory, including dried and thawed plasma and platelets (room temperature, cold‐stored, cryopreserved).	Authorities, regions, hospital boards, blood bank boards
Surge plans	Implement surge plan guidelines in blood banks and hospitals. Establish emergency whole blood collection in hospitals, remote locations and in military environment. Implement guidelines of required stock of material and reagents.	Authorities, regions, hospital boards, blood bank boards, military commanders
Harmonized procedures nationally and across Nordic countries	Implement harmonized procedures of emergency blood collection and testing. Implement guidelines of emergency blood transfusions, including prehospital blood transfusions.	Authorities, regions, hospital boards, blood bank boards
Formal agreements	Establish formal agreements for emergency blood support between regions and nations.	Authorities, regions, hospital boards, blood bank boards
Labelling/bar codes	Implement procedures for importing blood products in emergencies with new or unknown product codes.	Blood bank boards
Exemptions in emergencies	Harmonize and document permissible exemptions under SoHO art. 62‐67 (e.g., virus testing, non‐leucocyte depleted blood, transfusion of RhD positive red blood cells to females of childbearing age).	Authorities
Training and exercises	Conduct regular training on emergency routines in blood banks. Ensure annual participation of the blood banks in hospital emergency exercises. Include blood supply planning and military‐civilian cooperation in military exercises.	Blood bank boards Hospital boards Authorities and military commanders

Abbreviation: SoHO, Substances of Human Origin.

On the operational level, an extension of the programme for emergency collection and transfusion of WB from Walking Blood Banks is under development in Norway. Also, in Sweden and Finland, implementation of surge plans for emergency collection of WB is planned. Until now, the WB collection has primarily been planned from blood donors in blood banks. In the planning of blood collection outside established blood banks, approval from authorities and defined responsibilities are required. Training of personnel, maintaining competence, planning for material and equipment and recruitment of emergency donors must be organized [39].

Dried plasma products have the advantage of long shelf life, and they can be stored at ambient temperatures, making them ideal products for prehospital use [54]. Data suggest that reconstituted dried plasma transfusions can provide a survival benefit for prehospital transport times >20 min [36]. The low availability of dried plasma is a concern in the Nordic countries and worldwide, and the development and acquisition of dried plasma products has been a priority and is requested by the armed forces [55]. Dried plasma can be produced via lyophilization or spray‐drying techniques, using single or pooled donors and various pathogen inactivation methods [56]. Currently, dried plasma production is limited to a few centres—the German Red Cross and the French Armed Forces Blood Transfusion Centre (CTSA) [57, 58]. OctaplasLG Powder (Octapharma® Nordic AB, Stockholm, Sweden) is a recently approved pharmaceutical product available in the market [59]. New products and systems are under development; Velico® Medical, MA, USA, has developed a system for in‐house production of spray‐DP, under approval in the United States and Europe [60]. In Norway, a nationally funded development project for freeze‐dried plasma was initiated in 2022 [61]. Also in Sweden, the capability of national production of dried plasma has high priority and is requested by the armed forces. It is considered a strength to have different strategies in our three countries, as the shortage of plasma products and consumables for producing these is a potential risk.

### Training and exercises

We are working on improving training and exercises in both the civilian and the military. Training in hospitals, including the communication and collaboration between emergency departments and the transfusion services, is important and must be done regularly; identified needs for improvements should be documented and implemented. The laboratories should not only be involved in telephone chains but also in practical exercises with blood samples sent to the laboratory and requests for blood products [22]. Training should include manual routines and measures to manage cyber attacks and other disruptions of infrastructure, in addition to the use of alternative collection sites and systems for emergency collection of blood. It is important to include all stakeholders in planning and training. The supply of blood products should be included in the planning of military‐civilian exercises and in cross‐border military exercises. The procedures between our countries should be harmonized.

### Cross‐border harmonization of regulations and routines

On a strategic level, we work to identify and develop harmonized regulations regarding donor acceptance criteria, donor identification, quality criteria, required testing and traceability between our countries. The authorities must be involved in facilitating this. Cross‐border collaboration exists within prehospital care along national borders, allowing both ground ambulances and air ambulances to cross borders and provide treatment in neighbouring countries. This includes the administration of blood transfusions by air ambulance crews outside their home country, following their local clinical protocols. Treatment is subsequently reported to the receiving hospital upon patient handover. This practice must be formally agreed and documented, ensuring the traceability of the transfused blood.

### On‐going collaborative projects

The Nordic Health Preparedness agreement provides the basis for cooperation between the Nordic countries in crises and disasters. However, there has not been a Nordic plan for blood preparedness. In a project initiated and funded from Norway, the regions in the northern areas of Norway, Finland and Sweden are working on a cross‐border blood preparedness plan ‘Nordic Blood Preparedness Project’ [62]. These large areas have a low population density, but a large military footprint. The hospitals are small with a limited inventory of blood; the distances between existing infrastructure are large, and the numbers of air ambulances helicopters are low. This often results in delayed patient transportation. Often the distance to a hospital in a neighbouring country may be shorter than to an in‐country hospital. The blood supply and regulations for blood collection and transfusions need to be harmonized and traceability of the blood products ensured. The ‘Nordic Blood Preparedness Project’ includes using the procedures for emergency blood collection implemented in northern Norway as a model for a cross‐country interoperable blood preparedness system. Emergency collection and transfusion of WB will be implemented in rural hospitals and municipalities in the northern region.

## DISCUSSION

Bleeding is the most frequent cause of preventable deaths in civilian and war related trauma. Severe bleeding is also seen in non‐trauma related situations, such as gastrointestinal or post‐partum haemorrhage [63]. Early balanced blood transfusion, either with WB or with blood components, improves survival in patients with severe bleeding and is recommended in civilian and military guidelines [64, 65].

There are still gaps in national, regional and hospital blood preparedness plans. On the political level, we need agreements between the Nordic countries for mass casualty events for both civilian and military scenarios. We need to establish consensus on when and what exemptions to the regulations can be made in emergencies, in order not to delay the required blood support. Today this is poorly described, which increases the risk of regulatory delays in urgent situations.

Articles 62‐67 in the SoHO regulations concerning preparedness plans and actions in emergencies and armed conflicts should be interpreted and harmonized within the Nordic nations and with other EU nations [20].

The collection of LTOWB should be included in surge plans, at least in strategically positioned blood centres. Today only a few centres have implemented the use of WB transfusions in major bleeding, and mainly in prehospital services. Minimum requirements for blood and blood components in emergencies should be agreed upon and stated, for example, leucocyte reduction for WB, titre for LTOWB, use of RhD positive blood for women of childbearing age and if or when exemptions from virus testing can be made. All hospitals should have requirements for a minimal inventory, including blood stocks disposables and possibly shelf‐stable blood products, for example, dried plasma and cryopreserved platelets.

It is currently not feasible to make fresh blood products universally available in all settings. However, dried plasma may serve as a critical ‘bridge’ product for prehospital resuscitation in cases of severe bleeding [66]. In an observational prehospital study of standard care for resuscitation of bleeding patients in the Swedish ambulance service, it was shown that many of the bleeding patients have impaired coagulation and a high shock index (heart frequency/systolic blood pressure) at arrival at hospital after resuscitation with crystalloids [38]. Development efforts are on‐going for both pharmaceutical dried plasma products and in‐house production systems. Platelets are essential for haemostasis in bleeding patients, but their short shelf life of only 4–7 days presents logistical challenges [67]. Cryopreserved platelets offer a viable alternative with demonstrated safety and clinical effectiveness [48, 49]; however, in a recently published RCT, they were deemed inferior to fresh platelets, resulting in increased bleeding and more transfusions [52]. After thawing the recovery of cryopreserved platelets is approximately 70% and they exhibit phenotypic and functional changes such as reduced clot stability compared to fresh platelets, but they retain haemostatic function and are considered procoagulant and suitable for treating bleeding [47]. The defence forces request WB, primarily as LTOWB. Component therapy with WB equivalents (1:1:1 of RBCs, plasma and platelets) is considered suboptimal mainly for logistical reasons. According to NATO agreements and in existing Memorandum of Understanding (MOU), nations may be requested to provide blood support for NATO operations. Cross‐border collaboration and acceptance of blood products between nations within NATO and EU are prerequisites for multi‐national blood support in mass casualty events and in large‐scale combat operations.

Regular hospital exercises with active participation of the blood banks/transfusion services are important. Provision of type specific blood, safe routines for identity control and sampling for blood typing are required and must be exercised.

Blood group O RhD‐negative RBCs and WB are considered universal blood and used in emergencies. However, only 4%–6% of the blood donors in our countries are O RhD‐negative (internal data). In massive bleeding situations, it is not possible to supply only O RhD‐negative blood, and the stock of blood group O RBCs may also be limited. Guidelines and priorities are needed in those situations [68].

It is an ethical issue to aim for low numbers of expired and wasted blood, but at the same time always ensuring that blood is available for those in need. In unexpected events of bleeding, it may be a challenge to have sufficient blood available in the right place. To mitigate this, we need blood products with longer shelf life. Dried plasma that can be stored for up to 24 months and cryopreserved platelets stored for several years can be made immediately available and are important as a backup.

Although research of haemoglobin solutions, artificial RBCs, lyophilized platelets, universal red cells and cultured cells is on‐going, these products are still far from being available for routine human use.

### Limitations

The blood preparedness plans in the Nordic countries may, or may not, be directly applicable for other countries with different regulations, organizational structure and geographical conditions. Nevertheless, emergency preparedness plans are required in all countries. Donor planning and maintaining a sustainable supply are crucial but not discussed in this article with focus on timely blood supply in emergencies. All future plans described are not finalized and implemented and may not be identical in our three countries. In addition to the treatment of haemorrhage, plasma products are also essential in the management of burn injuries, due to their role in restoring plasma volume and supporting coagulation. Furthermore, in the event of nuclear incidents, there may be a need for platelets and haematopoietic stem cells to treat radiation‐induced bone marrow suppression. These aspects, however, are not addressed in the current manuscript, but should be included in preparedness plans.

There are more on‐going preparedness projects in the health care sector, which have not been discussed here, as well as other projects with higher security classifications.

## CONCLUSION

Availability of blood product as close as possible to bleeding patients has been shown to save lives. To accomplish this in our countries with large rural areas and small hospitals, we need shelf‐stable blood products and surge plans that can be timely activated. Cross‐border blood preparedness plans for disaster and war are required, and blood supply must be included in hospital and military mass casualty exercises.

## CONFLICT OF INTEREST STATEMENT

The authors declare no conflicts of interest.

## Data Availability

The data that support the findings of this study are available from the corresponding author upon reasonable request.
